# Nutritional and bioactive characteristics of buckwheat, and its potential for developing gluten‐free products: An updated overview

**DOI:** 10.1002/fsn3.3166

**Published:** 2022-12-22

**Authors:** Sajad Ahmad Sofi, Naseer Ahmed, Asmat Farooq, Shafiya Rafiq, Sajad Majeed Zargar, Fozia Kamran, Tanveer Ali Dar, Shabir Ahmad Mir, B. N. Dar, Amin Mousavi Khaneghah

**Affiliations:** ^1^ Department of Food Technology Islamic University of Science & Technology Awantipora Jammu and Kashmir India; ^2^ Department of Food Technology DKSG Akal College of Agriculture Eternal University Baru Sahib Himachal Pradesh India; ^3^ Division of Biochemistry Sher‐e‐Kashmir University of Agricultural Sciences and Technology of Jammu Chatha Jammu and Kashmir India; ^4^ Proteomics Laboratory, Division of Plant Biotechnology Sher‐e‐Kashmir University of Agricultural Sciences and Technology of Kashmir Shalimar Jammu and Kashmir India; ^5^ School of Science, Parramatta Campus Western Sydney University Penrith New South Wales Australia; ^6^ Department of Clinical Biochemistry University of Kashmir Hazratbal, Srinagar India; ^7^ Department of Food Science & Technology Govt. College for Woman Srinagar India; ^8^ Department of Fruit and Vegetable Product Technology Prof. Wacław Dąbrowski Institute of Agricultural and Food Biotechnology – State Research Institute Warsaw Poland

**Keywords:** bioactive, celiac diseases, flavonoids, gluten‐free, nutraceutical

## Abstract

In the present era, food scientists are concerned about exploiting functional crops with nutraceutical properties. Buckwheat is one of the functional pseudocereals with nutraceutical components used in the treatment of health‐related diseases, malnutrition, and celiac diseases. As a preferred diet as a gluten‐free product for celiac diseases, buckwheat is a good source of nutrients, bioactive components, phytochemicals, and antioxidants. The general characteristics and better nutritional profile of buckwheat than other cereal family crops were highlighted by previous investigations. In buckwheats, bioactive components like peptides, flavonoids, phenolic acids, d‐fagomine, fagopyritols, and fagopyrins are posing significant health benefits. This study highlights the current knowledge about buckwheat and its characteristics, nutritional constituents, bioactive components, and their potential for developing gluten‐free products to target celiac people (1.4% of the world population) and other health‐related diseases.

## Graphical abstract

Buckwheat is one of the functional pseudocereals with nutraceutical components to target health‐related diseases, malnutrition, and celiac. In recent years, buckwheat‐related food products with good sensory and techno‐functional qualities attractive the food market with health benefits and suitable food for people with gluten intolerance. Incorporating buckwheat in product formulation would help mitigate various health‐related problems and gluten‐free products with nutrient‐dense ingredients.

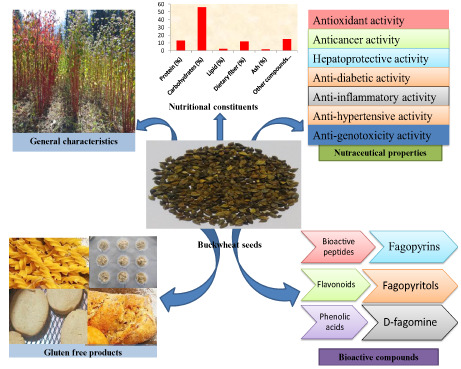

## 1 INTRODUCTION

In recent years, the consumption of functional food with bioactive ingredients has increased in consumers' diets. These functional foods provide nutritional as well as health benefits to end‐use consumers. Among the health‐related foods, pseudocereals are among the functional foods with numerous health benefits (Astrini et al., 2020; Mir et al., 2018; Xu et al., 2022). However, pseudocereals are taxonomically different; they show similar characteristics to the Poaceae family (wheat, rice, and barley) due to endosperm‐rich starch components. The main pseudocereals with health‐related benefits are buckwheat, amaranth, and quinoa (Ferreira et al., 2022). Buckwheat is one of the pseudocereals, belongs to the family of Polygonaceae, and is commonly used in the cold region of the world. The buckwheat cultivars are mainly found in mountain regions, especially Russia and China (Begemann et al., 2021; Yilmaz et al., 2020; Zou et al., 2021). The world production of buckwheat is about 3.8 million tons, and Russia ranked at the top position with 1.5 million tons, followed by China with 0.9 million tons (FAO STAT data, 2019). Buckwheat is also cultivated in France (8.3%), the USA (5.7%), Poland (5.4%), Brazil (3.5%), and Japan (1.0%). Buckwheat seeds are mainly used as breakfast cereals in the form of groats, flour for bakery products, and other enriched products such as bread, tea, honey, and sprouts (Giménez‐Bastida et al., 2015; Małgorzata et al., 2018). The various health‐related benefits (hypocholesterolemic, hypoglycemic, anticancer, and anti‐inflammatory) were associated with buckwheat and its byproducts which enhance their potential for functional food formulation (Mondal et al., 2021) and increase their agricultural, industrial, and pharmaceutical uses (Fotschki et al., 2020).

The buckwheat's bioactive components, such as proteins, dietary fiber, vitamins, flavonoids, fagopyrins, d‐fagomine, and phenolic acids, have good healing properties against chronic diseases (Zhou, Hao, et al., 2015; Zhou, Wen, et al., 2015). Buckwheat seeds are a good source of proteins with well‐balanced amino acids and contain albumins, globulins, prolamins, and glutelins (Jin et al., 2022). The buckwheat proteins are free from gluten, increasing their acceptability by people suffering from celiac diseases (Bobkov, 2016). Proteins in buckwheat are a rich source of leucine, phenylalanine, lysine, threonine isoleucine, cysteine, and asparagine (Bhinder et al., 2020). The biological values of buckwheat proteins are outstanding, but antinutritional factors (tannins and proteases) associated with buckwheat proteins lower their protein digestibility (Mattila et al., 2018). Polyphenolic compounds (flavonoids and phenolic acids) are bioactive ingredients in buckwheat and increase the nutraceutical potential of buckwheat. Buckwheat is a rich source of flavonoids such as rutin, isoorientin, quercetin, isovitexin, vitexin, and orientin (Raguindin et al., 2021). Among all pseudocereals, rutin is only present in buckwheat, with higher antioxidant, anti‐inflammation, and anticancer properties (Zhu, 2016). The flavonoid compounds in buckwheat impart pharmaceutical and other health‐related benefits (Lee et al., 2016). Buckwheat is also a good source of resistant starch, tannins, plant sterols, and fagopyrins (Ahmed et al., 2014).

There are two well‐known disorders linked to gluten exposure: celiac disease and IgE‐mediated wheat allergy. Genetic susceptibility factors play a significant role in the development of celiac disease, an autoimmune condition that may cause significant intestine damage. In the general community, celiac disease affects 0.5% to 1% of people (Giménez‐Bastida et al., 2015; Manikantan et al., 2022; Rafiq et al., 2021). Contrarily, gluten sensitivity and/or other wheat proteins is caused by IgE antibodies that identify epitopes from certain proteins, known as allergens, setting off a chain of events that results in allergic inflammation. Wheat allergy is present in between 0.33% and 1.17% of the population (Ballini et al., 2021; Brand et al., 2022; Srisuwatchari et al., 2020). A rigorous, gluten‐free diet for the rest of one's life is only suggested for those diagnosed with celiac disease. Similar restrictions apply to those who have been diagnosed with IgE‐mediated wheat allergy. In that context, nonceliac gluten sensitivity, a third illness marked by discomfort after ingestion of gluten and in which neither celiac disease nor IgE‐mediated allergy plays a role, has received more attention in recent years (Aksoy et al., 2021; Srisuwatchari et al., 2020; Vassilopoulou et al., 2021). Various products without gluten protein are available on the market, but most products lack acceptance by celiac patients (Mir et al., 2014). Products free from gluten are available in the market with low protein, dietary fiber, and vitamins compared to gluten‐containing food products. So, designing through fortification or supplementation of nutrient‐dense ingredients is the novel approach for enhancing the nutritional profile of gluten‐free products. Buckwheat is a nutrient‐dense pseudocereal, free from gluten protein, and the preferred diet for celiac patients (Morales et al., 2021). This study highlights the current knowledge about buckwheat and its characteristics, nutritional constituents, bioactive components, and potential for developing gluten‐free products.

## 2 DIVERSITY AND GROWING ASPECTS OF BUCKWHEAT

**FIGURE 1:**
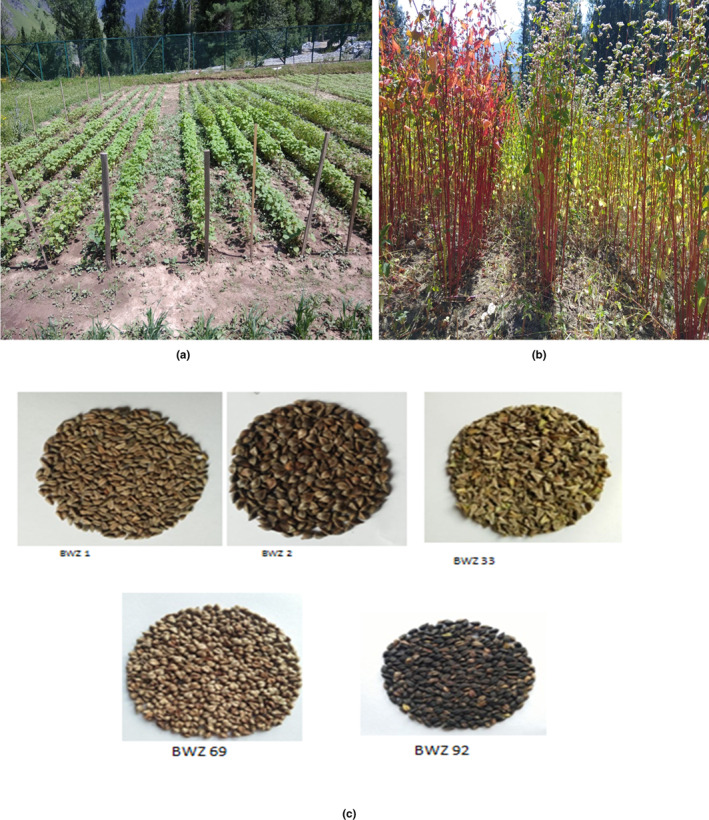
Different lines/selections of buckwheat (a), buckwheat at harvesting stage (b), and seeds from different lines from buckwheat (c).

Buckwheat is an annual herbaceous plant adaptable to all environmental conditions, including infertile land unsuitable for other crops (Rodríguez et al., 2020). The origin of buckwheat is China and Central Asia (Zhang et al., 2012). Buckwheat is derived from beech and wheat due to its similarity with beechnut and wheat. Buckwheat has been grouped into annual and multiannual types of species. The annual species includ*e Fagopyrum tataricum* L, *Fagopyrum giganteum* Krotov, and *Fagopyrum esculentum* Moench, whereas *Fagopyrum suffruticosum* Fr. Schmidt, *Fagopyrum ciliatum* Jaegt, and *Fagopyrum cymosum* Meissn are multiennual types (Jing et al., 2016). The *Fagopyrum esculentum* and *Fagopyrum tataricum* are common, and Tartary buckwheat types are most widely grown in the Himalayan regions. The buckwheat cultivars or lines with the harvesting stage and different varieties of seeds from buckwheat cultivars are presented in Figure 1. Buckwheat has been called different names by different countries, such as ogal (India), grecicha kulfurnaja (Russia), sarrasin (French), mite phapar (Nepal), Soba (Japan), Jawas (Pakistan), poganka (Poland), buchweizen (Germany), and fagopiro (Italy) (Ahmed et al., 2014).

The buckwheat has a diversified crop ecology depending upon season and climate. Buckwheat is a mostly adapted crop of the Himalayas at different altitudes, such as low hills, mid‐hills, and high hills. The most commonly grown buckwheat is adapted to low hills and is a winter crop, while tartary buckwheat is suitable for mid‐ and high hills and is a spring‐growing crop (Koirala, 2020). Buckwheat is a dicot with irregular and triangular seeds (Ahmed et al., 2014). The seed contains brown‐to‐black color hulls covering the whole kernel, colored white to light green. The color and hardness of buckwheat hull are related to different cultivars of buckwheat (Roy et al., 2019). The buckwheat seed germinates in soil (3–4 days), flowering after 3 weeks of planting with white petals, and after pollination by wind, seed formation starts within 10 days. The buckwheat seed needs more than 1 week to attain full maturity after seed formation. The buckwheat seed for growth requires low‐fertility soil with a moderate nitrogen content (Gairhe et al., 2015). The cultivation period of buckwheat is a short growing period of 70–90 days and good storage periods due to its chemical constituents. The harvesting of buckwheat seeds has technical problems due to their uneven ripening pattern. So modern techniques and harvesting equipment need to be adopted to tackle the problems and issues related to the harvesting of buckwheat.

## 3 NUTRITIONAL CONSTITUENTS OF BUCKWHEAT

Buckwheat is a nutrient‐dense grain with an excellent nutrient profile to target malnutrition and the celiac population. The buckwheat type pseudocereal is preferred for formulating food products through supplementation with cereal crops to enhance nutritive value or replace cereal grains with gluten‐free product formulation. The nutritional composition of buckwheat is presented in Table 1.

**TABLE 1:** Nutritional composition of buckwheat

Parameters	General^a^	Cultivars^b^	
		Common buckwheat	Tartary buckwheat
Protein (%)	13.07	12.30	13.15
Carbohydrates (%)	56	54.50	57.40
Lipid (%)	2.52	3.80	3.84
Dietary fiber (%)	11.94	7.0	10.60
Ash (%)	1.67	2.0	2.70
Other compounds (%) (soluble carbohydrates, phenolic cpds, organic acid, nucleotides, and other unknown cpds)	14.80	18.40	10.53

Abbreviations: cpds, compounds.

^a^ Adopted and modified from Collar & Angioloni, (2014).

^b^ Adopted and modified from Antonio et al., (2015).

### 3.1 Proteins

Buckwheat is an important source of protein content (8.5%–18.8%), depending on the cultivar, source, and climate conditions (Dziadek et al., 2016). The protein concentration in buckwheat grains is higher than in cereal grains (Bobkov, 2016). The proteins in buckwheat are composed of globulins (43.3%–64.5%), albumins (12.5%–18.2%), prolamins (0.8%–2.9%), and glutelins (8.0%–22.7%), and 15% of residual proteins (Chrungoo et al., 2016). The prolamine content in buckwheat proteins is very low, and proteins responsible for celiac diseases (30 kDa prolamins) are absent in buckwheat as observed from gel electrophoresis and enzyme‐linked immunosorbent method by Petr et al. (2003). The 2 S albumins, 8 S, and 13 S globulins in buckwheat proteins are similar to the storage proteins of legumin (Bobkov, 2016). Among the globulin proteins, 13 S globulins are the main storage protein having a hexameric structure with acidic (32–43 kDa) and basic (23–25 kDa) polypeptide subunits bonded by disulfide bonding (Taylor et al., 2016). The compositions of amino acids are well balanced in buckwheat proteins and are rich in arginine, lysine, and aspartic acids (Bhinder et al., 2020). The presence of tannins and protease inhibitors decreases the protein digestibility of buckwheat. However, the presence of lysine amino acid in buckwheat protein increases the protein digestibility‐corrected amino acid scores of proteins in buckwheat than the cereals (Zhang et al., 2012).

### 3.2 Carbohydrates

Starch is the available carbohydrate source in buckwheat grains that varies from 60% to 70% (Vojtiskova et al., 2012). The amylose and amylopectin are in the ratio of 25 and 75%, respectively, present in the starch of buckwheat (Qin et al., 2010). Buckwheat starch granules are small in size, smooth surfaced, polygonal in shape and size (3–10 μm), and are similar to tuber and cereal starch (Yang et al., 2019). Buckwheat is also a good source of resistant starch (33.5%) as Yang et al. (2019) reported in buckwheat groats. Different processes, such as autoclaving, cooking, or boiling, affect buckwheat‐resistant starch content (Bobkov, 2016). Buckwheat contains more starch content than the other pseudo cereals, and the calorie content (343 cal/100 gm) of buckwheat is similar to cereal and legumes (Mir et al., 2014). The buckwheat embryo is a storehouse of soluble carbohydrates in the form of d‐chiro‐inositol, besides sucrose. The d‐chiro‐inositol in the embryo of buckwheat is stored in the form of fagopyritols which is galactosyl derivative of d‐chiro‐inositol, and their concentration varied from 20.7 to 41.7 mg 100/g, out of which 71% is concentrated in buckwheat embryo (Zieliński et al., 2019). The maximum concentrations of fagopyritols are reported in Tartary buckwheat than in the common cultivars. Another soluble carbohydrate has been identified from tartary buckwheat as rhamnosyl glucoside (31%) (Dębski et al., 2016).

### 3.3 Dietary fiber

The dietary fiber is the main component in the proximate composition of buckwheat, higher than other pseudocereals and similar to cereal grains (Alvarez‐Jubetea et al., 2010). Dietary fiber content varies in different processed fractions of buckwheat, with 23.8% for unhusked, 10.3% for husked, and 7% for groats, and their concentration varies between the cultivars (Qin et al., 2010). The dietary fiber is mainly concentrated in the seed coat of the buckwheat, which, after the milling process, reduces the fiber content (Wefers & Bunzel, 2015). The dietary fiber is classified into soluble (pectin and gums) and insoluble (lignin and cellulose) dietary fibers. The soluble dietary fiber of 4.8% and insoluble dietary fiber of 2.2% are present in buckwheat, with health effects against cholesterol and obesity, and also induce adverse effects such as mineral and protein unavailability (Zhu, 2020).

### 3.4 Lipids

The lipids in buckwheat are low but have shown good importance in various physiological activities, with lipid content ranging from 1.5% to 3.7% (Ruan et al., 2020). The lipids in buckwheat are classified into neutral lipids at 81–85%, phospholipids at 8%–11%, and glycolipids at 3%–5% (Bobkov, 2016). Buckwheat is a rich source of unsaturated fatty acids (74.5%–79.3%), which have health benefits against heart diseases, cancer, inflammation, and diabetes (Ruan et al., 2020). The unsaturated fatty acids are concentrated in the embryo of buckwheat seed, and palmitic, oleic, and linoleic are the most common type of fatty acids, representing 87.3%–88% in buckwheat seeds (Gulpinar et al., 2012).

### 3.5 Vitamins and minerals

Vitamins and minerals are leading in physiological processes in the human body. The buckwheat grain is a rich source of vitamin A, vitamin B complexes, and vitamins C and E (Zhu, 2016). The thiamine, riboflavin, niacin, pantothenic acid, and pyridoxine are vitamin B complexes in a concentration of 0.22, 0.1, 1.8, 1.1, and 0.17 mg/100 g (Ahmed et al., 2014). The vitamin E (tocopherol) in the buckwheat grains as the natural antioxidant varies in concentration to 140.1 μg/g (Zhou, Hao, et al., 2015; Zhou, Wen, et al., 2015). Vitamin B1 of buckwheat is associated with thiamine‐binding proteins and increases their bioavailability and stability during storage conditions (Wronkowska, Zielinska, et al., 2010; Wronkowska, Soral‐Śmietana, et al., 2010). Zhou, Hao et al. (2015) and Zhou, Wen et al. (2015) reported a vitamin C content of 5 mg/100 g in buckwheat, and after germination, the vitamin C levels rose to 25 mg/100 g. Buckwheat grains are mineral sources of both macro‐ and micronutrients. Macronutrients like phosphorus, potassium, magnesium, and calcium are at reasonable levels, whereas iron, manganese, and zinc are at lower concentrations in buckwheat (Zhu, 2016). The micronutrients in buckwheat are higher than in cereal grains, and their concentration is mainly confined to the seed coat, hull, and aleurone layers (Orožen et al., 2012). The minerals like zinc, copper, and potassium become readily available for absorption after enzymatic digestion into a soluble form (Klepacka et al., 2020). The diet available for gluten‐free consumers is low in vitamins and minerals, and the incorporation of buckwheat in gluten‐free diets increases the concentration of vitamins and essential minerals in their diet (Mir et al., 2014).

## 4 BIOACTIVE COMPOUNDS IN BUCKWHEAT

**FIGURE 2:**
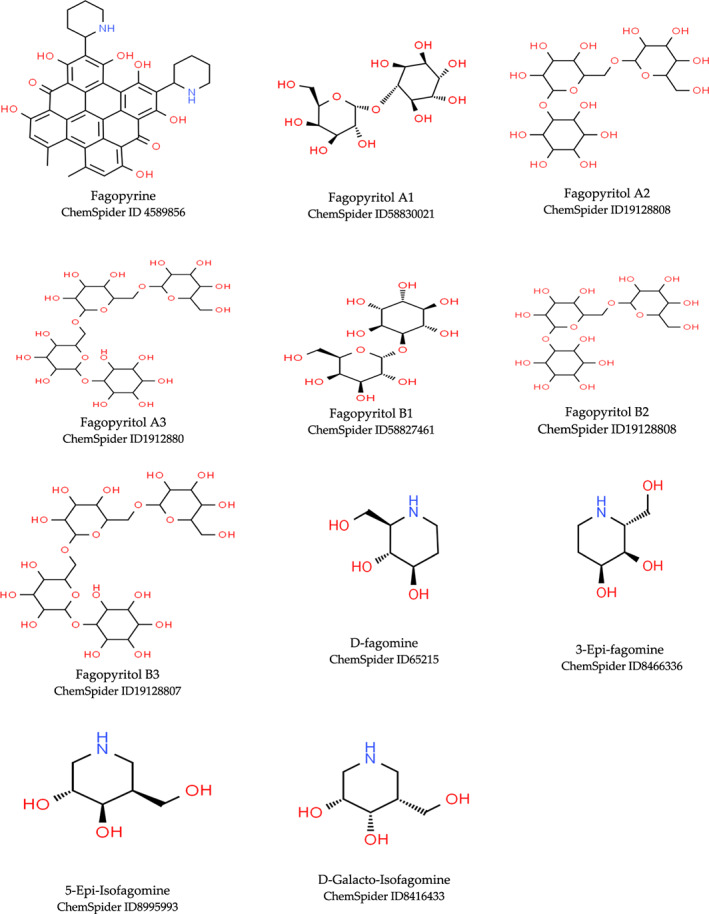
Chemical structure of fagopyrins, fagopyritols, and d‐fagomine‐based bioactive compounds in buckwheat

Buckwheat is a dense nutritive pseudocereal with various bioactive compounds such as bioactive peptides, flavonoids, fagopyrins, fagopyritols, d‐fagomine, and phenolic acids (Table 2), and their chemical structure is presented in Figure 2. The bioactive compound of buckwheat grains enhances its healing effect against health‐related diseases.

**TABLE 2:** Bioactive components of buckwheat

Class	Types	Examples	References
1. Bioactive peptides	Antimicrobial peptides	Fa‐AMP1	Fujimura et al. (2003)
		BWI‐1	Belozersky et al. (1995)
		BWI‐2c	Oparin et al. (2012)
		FtTI	Ruan et al. (2011)
	Trypsin inhibitors	BWI‐1	Begemann et al. (2021)
		BTI‐1	Oparin et al. (2012)
		BWI‐2a	Belozersky et al. (1995)
		BWI‐2b	Dunaevsky et al. (2004)
		BWI‐2c	Ruan et al. (2011)
		BWI‐4a	
		BWI‐4c	
		FtTI	
	Antitumor proteins	rBT1	Li et al. (2009)
		BWI‐1 & BWI‐2a	Park and Ohba (2004)
		TBWSP31	Guo et al. (2011)
	Hypotensive peptides	ACE inhibitory peptides (FY, AY, LF, YV, VK, YQ, YQY, PSY, LGI, ITF, and INSQ).	Li et al. (2002)
		BPL peptides (DVWY, FDART, FQ, VAE, VVG, and WTFR)	Koyam et al. (2013)
	Antidiabetic peptides	Antioxidant proteins (WPL, VPW, VFPW, and PW)	Ma et al. (2006)
2. Flavonoids	Rutin, orientin, vitexin, quercetin, isovitexin, and isoorientin		Lee et al. (2012)
3. Polyphenol compounds	Fagopyrins		Ahmed et al. (2014)
	Phenolic acids (p‐hydroxybenzoic, ferulic, protocatechuic, p‐coumaric, gallic, caffeic, vanillic, chlorogenic, syringic, and salicylic acids).		Guo et al., 2011), Sytar (2015)
4. Carbohydrates	Fagopyritols (A1, A2, A3, B1, B2, and B3)		Steadman et al. (2000)
5. Imino sugars	d‐fagomine		Amezqueta et al. (2012)

Abbreviations: AC, angiotensin I‐converting enzyme; BP, blood pressure lowering.

### 4.1 Bioactive peptides

Buckwheat grains are a good source of protein higher than cereal crops (Bobkov, 2016). The protein content in buckwheat has well‐balanced amino acids with a nutritive value similar to milk and egg solids (Gimenez‐Bastida & Zielinski, 2015; Zhou, Hao, et al., 2015; Zhou, Wen, et al., 2015). According to Clare and Swaisgood (2000), bioactive peptides have pharmacological activity. The pharmacological activities associated with bioactive peptides are antioxidant, antimicrobial properties, cholesterol‐lowering ability, hypoglycemic effect, and antitumor activity (Nasri, 2016). The peptide generally contains 3 to 50 amino acid residues, with their activity depending on their sequence, composition, structure, charge, and hydrophobicity (Saadi et al., 2015). Bioactive peptides are either naturally available or prepared through enzymatic hydrolysis (Aiello et al., 2017). Buckwheat is a source of antimicrobial, trypsin inhibitors, antitumor proteins, and hypotensive, and antidiabetic peptides with numerous health benefits (Gimenez‐Bastida & Zielinski, 2015; Zhou, Hao, et al., 2015; Zhou, Wen, et al., 2015).

Antimicrobial peptides are the natural plant defenses isolated from buckwheat seed and bran with antimicrobial properties against various pathogens (Bai‐Ngew et al., 2021; Zhou, Hao, et al., 2015; Zhou, Wen, et al., 2015). The buckwheat antimicrobial peptide contains 45–55 amino acid sequences and four disulfide bridges, having eight disulfide‐linked cysteines residues (Thomma et al., 2002). The buckwheat antimicrobial peptides such as Fa‐AMP1, Fa‐AMP2, BWI‐1, BWI‐2c, and FtTI have been isolated with activity against fungi, gram‐negative bacteria, and gram‐positive bacteria (Belozersky et al., 1995; Fujimura et al., 2003; Oparin et al., 2012; Ruan et al., 2011). The antifungal peptides Fa‐AMP1 and Fa‐AMP2 are effective against the growth of *Geotrichum candidum*, *Fusarium oxysporum*, and *Mycosphaerella arachidicola* (Fujimura et al., 2003). BWI and BWI‐2c are the buckwheat‐based protease inhibitor that inhibits the growth of *Alternaria alternate*, *Fusarium oxysporum*, *Pseudomonas syringae*, and *Clavibacter michiganensis* (Khadeeva et al., 2009). FtTI are the low molecular weight antifungal peptide (14 kDa) isolated from the tartary buckwheat cultivar with anti fungal activity against *Colletotrichum glaeosporioides, Phytophthora capsici, Alternaria cucumerina*, and *Alternaria solani* at 30 μg/mL of concentration (Ruan et al., 2011).

Buckwheat trypsin inhibitor inhibits trypsin and chymotrypsin enzyme activity and lowers the bioavailability of buckwheat proteins. Trypsin inhibitors are endogenous antinutritional factors that limit the digestibility of proteins by affecting their absorption. Buckwheat trypsin inhibitors, besides limiting digestibility, have health benefits against the growth of cancer cells, inhibiting reverse transcriptase of HIV‐1 and bacterial origin proteinase (Zhou, Hao, et al., 2015; Zhou, Wen, et al., 2015). The trypsin inhibitors isolated from buckwheat were BWI‐1, BTI‐1, BWI‐2a, BWI‐2b, BWI‐2c, BWI‐4a, BWI‐4c, and FtTI (Zhou, Hao, et al., 2015; Zhou, Wen, et al., 2015). Buckwheat antitumor proteins are protease inhibitors that affect the transport of protease toward the surrounding organs and tissues (Zhou, Hao, et al., 2015; Zhou, Wen, et al., 2015). The protease inhibitors (BWI‐1 and BWI‐2a) were reported by Park and Ohba (2004) as antitumor proteins on human T‐ALL cell lines that activated tumor cell apoptosis by segmenting cell DNA. Recombinant buckwheat trypsin inhibitor (rBTI) tested on EC9706, HepG2, and HeLa cell lines induce cell apoptosis through the up‐ and downregulation of Bax and Bak, and Bcl‐2 and Bcl‐xl, respectively, by the mitochondrial apoptotic pathway (Li et al., 2009). The assay of MTT and cytometry showed the inhibitory effect of rBTI in IM‐ 9 and HL‐60 cells (Gao et al., 2007; Zhang et al., 2007). The antitumor protein TBWSP31 inhibits the growth of Bcap37 human mammary cancer cell lines by up‐ and downregulation of Fas and bcl‐2, respectively (Guo et al., 2010). Buckwheat‐based protein extracts have also been effective against dimethylhydrazine‐induced mammary and colon cancer in rats (Liu et al., 2001).

Buckwheat hypotensive peptides contain 2–5 amino acid residues (Zhou, Hao, et al., 2015; Zhou, Wen, et al., 2015). The hypotensive peptides inhibit the angiotensin I‐converting enzyme and lower blood pressure (Zieliński et al., 2020). The hypotensive peptides with amino acids (proline, tyrosine, tryptophan, tyrosine, proline, and phenylalanine) at the carboxyl and terminal end have a great affinity for inhibiting the activity of the angiotensin I‐converting enzyme (Ma et al., 2006). The inhibitors of angiotensin I‐converting enzyme isolated from protein hydrolysates of buckwheat were FY, YQ, AY, LF, YQY, YV, VK, PSY, LGI, ITF, and INSQ (Li et al., 2002). The inhibitors such as DVWY, FDART, FQ, VAE, VVG, and WTFR were also reported by Koyam et al. (2013) in fermented buckwheat sprouts with hypotensive activity. The buckwheat protein extracts have been reported as the antidiabetic property (Zhou, Hao, et al., 2015; Zhou, Wen, et al., 2015). The reactive oxygen species generated during metabolic processes damage the pancreatic islets' cell membrane, which is one of the reasons for diabetes (Fakhruddin et al., 2017). The damage of pancreatic islets can be reduced by the intake of buckwheat proteins containing antioxidant enzymes and reactive oxygen species scavenging activity (Liu et al., 2009). Buckwheat proteins maintain the balance of glucose levels in diabetic people (Zhou, Hao, et al., 2015; Zhou, Wen, et al., 2015). Buckwheat‐digested proteins produce antioxidant peptides (WPL, VPW, VFPW, and PW) with a strong scavenging capacity of reactive oxygen species (Zhou, Hao, et al., 2015; Zhou, Wen, et al., 2015). Buckwheat proteins are an effective supplement for people suffering from high cholesterol levels and obesity. Buckwheat protein extracts can bind with bile acids in the gastrointestinal tract, which increase the neutral sterols excretion through feces (El‐Sayed et al., 2020). The binding affinity of buckwheat protein extract with bile acid increases the secretion of liver bile acid from cholesterol and lowers the liver cholesterol level (Zhou, Hao, et al., 2015; Zhou, Wen, et al., 2015).

### 4.2 Flavonoids

Flavonoids are naturally occurring polyphenolic compounds with a phenyl benzopyrone structure (Ahmed et al., 2014; Karami et al., 2021). These phenolic compounds have various functional and biological properties. Based on phenylbenopyrone structure (saturation level and pyran ring), flavonoids are classified into flavones, flavonols, isoflavones, flavonols, flavanones, and flavanonols (Borovaya & Klykov, 2020; Kaşıkçı & Bağdatlıoğlu, 2022). The flavonoids identified from buckwheat groats and hulls are rutin, orientin, vitexin, quercetin, isovitexin, and isoorientin (Zhang et al., 2012). Rutin is an important flavonoid compound found only in buckwheat among the pseudocereals (Das et al., 2019). Rutin is a glycoside of quercetin with rutinose as a carbohydrate moiety and chemically denoted as quercetin‐3‐beta‐d‐rutinoside. The rutin content is accumulated in different regions of the buckwheat plant, such as inflorescence, stalks, leaves, and grains (Ahmed et al., 2014). The maximum level of rutin content is present in leaves (0·08–0·10 mg/g), and in buckwheat seeds, it varied from 0·12–0·36 mg/g (Brunori et al., 2010; Park et al., 2008). The therapeutic and biological activities associated with rutin content increase the elasticity of blood vessels, effectiveness against circulatory problems, atherosclerosis, antioxidant property, anti‐inflammation, antihypertension, vasoconstrictive, spasmolytic, UV‐light protectant, decrease cholesterol, and protect against oxidative stress and gastric lesions (Ahmed et al., 2014; Gong et al., 2010; Kuntic et al., 2011; Landberg et al., 2011; Mukasa et al., 2009). The other flavonoids besides rutin in buckwheat are hyperoside, quercitrin, and catechins (Kalinova & Vrchotova, 2009).

### 4.3 Fagopyrins

Fagopyrin is a photosensitive polyphenol compound present in buckwheat seeds. The fagopyrins have a naphthodianthrone skeleton with a structure similar to hypericin compounds (Kim & Hwang, 2020). Fagopyrins are considered a toxic polyphenol due to their photosensitization and allergy reaction present in seeds and leaves of buckwheat. They are difficult to isolate due to their lower content in buckwheat (Ahmed et al., 2014). The fagopyrins in buckwheat have laxative, antibiotic, and antiviral effects and treatment for diabetes (Hagels, 2007).

### 4.4 Fagopyritols

Fagopyritols are d‐chiro‐inositol with galactosyl derivatives present in bran fraction and higher concentration in buckwheat embryos. d‐chiro‐inositol is an isomer of inositol accumulated in buckwheat seeds in the form of fagopyritols (Cheng et al., 2019). Fagopyritols are water‐soluble carbohydrates (71%) localized in the embryo of buckwheat seed (Bobkov, 2016) and prevent buckwheat plants from desiccation during seed development. The various types of fagopyritols, such as A1, A2, A3, B1, B2, and B3, are identified in buckwheat seeds with different bonding positions, and type B1 is the most common and abundant soluble carbohydrates (41.16%) (Ueda et al., 2005). Germination treatment of buckwheat seeds increases the concentration of soluble carbohydrates, mostly fagopyritols. The increase in the concentration of fagopyritol during germination is temperature dependent, with fagopyritol B1 increasing at 18°C and fagopyritol A2 and B2 at 25°C (Bobkov, 2016). The effects of fagopyritols in natural buckwheat enhance its nutraceutical and pharmaceutical potential against type II diabetes, polycystic ovaries development, and plasma cholesterol (Khalid et al., 2020).

### 4.5 d‐fagomine

d‐fagomine is an iminosugar associated with buckwheat (groats, bran, leaves, and flour) as a dietary food component with biological activity and antimicrobial properties (Amezqueta et al., 2012). The d‐fagomine is chemically a polyhydroxylated piperidines compound and acts as an inhibitor against the glycosidase enzyme (Masdeu et al., 2021). d‐fagomine and its isomer 3,4‐di‐epi‐fagomine have been reported in buckwheat with higher concentration (43–44 mg/kg) in groats (Amezqueta et al., 2012). d‐fagomine is a pharmaceutical property against diabetes, pathogenic diseases, cancer, AIDS, overweight, and viral diseases (Amezqueta et al., 2012).

### 4.6 Phenolic acids

Buckwheat is the richest source of phenolic acids available in free or bound form (Li et al., 2016). The phenolic acid in buckwheat is mostly benzoic acid and cinnamic acid derivatives (Mir et al., 2018). The phenolic acids in buckwheat are p‐hydroxybenzoic, syringic protocatechuic, vanillic, ferulic, p‐coumaric, gallic, caffeic, chlorogenic, and salicylic (Guo et al., 2011; Sytar, 2015; Xiong et al., 2022). Phenolic acids in buckwheat are associated with antioxidant activity (Gimenez‐Bastida & Zielinski, 2015) and prevent the buckwheat seed from chemical degradation during prolonged storage (Antoniewska et al., 2018). Phenolic acids are natural antioxidants, effective against reactive oxygen species, reducing cardiovascular diseases, cancer, and age‐related processes (Forni et al., 2019; Wani et al., 2022). The phenolic acids from buckwheat bran have biological activity against liver cancer cells, as tested in vitro by Li et al. (2016).

## 5 HEALTH‐PROMOTING ATTRIBUTES OF BUCKWHEAT

Buckwheat is a potentially promising herb used in ancient times to treat health‐related diseases (Cai et al., 2016). Buckwheat is pseudocereal rich in protein and starch content, with a rich source of minerals and vitamins (Khalid et al., 2020). The unique nature, structure, and bioactive compounds of buckwheat have a vast potential to support nutraceutical potential to human health (Zhu, 2016). Buckwheat provides a good source of nutrients and bioactive components, resulting in the enhancement of the therapeutic potential of buckwheat. The therapeutic potential of buckwheat is as follows:

### 5.1 Antioxidant activity

The antioxidant activity of buckwheat is due to the presence of polyphenolic compounds, particularly rutin content. Due to the generation of reactive oxygen species in human metabolism, free radicals are responsible for cancer, cardiovascular disease, aging, cerebrovascular disease, and degenerative diseases. Additionally, it has been shown that naturally occurring polyphenolic antioxidants decreased the ROS in antigen‐IgE‐activated mast cells and concurrently suppressed the release of histamine by these activated mast cells. Therefore, it may be hypothesized that buckwheat's antiallergic effects are partially a result of its polyphenolic components' antioxidant properties (Ahmed et al., 2014; Papadopoulou et al., 2021; Ünsal et al., 2021). However, more research must be done to determine how buckwheat and its components affect allergic responses. Buckwheat flavonoids can act as scavengers against free radicals due to their ease of oxidation (Zhou et al., 2018). Buckwheat flavonoids have a molecular structure that supports the concept of an active phenolic hydroxyl group capable of scavenging free radicals and preventing cancer, cardiovascular disease, aging, and cerebrovascular and degenerative diseases (Li et al., 2017; Shahbaz et al., 2022).

### 5.2 Antiallergic activity

Buckwheat (*F. esculentum*) grain extract (BGE) substantially decreased compound 48/80‐induced vascular permeability as measured by Evans blue extravasation, whether administered orally, intraperitoneally, or intradermally. Oral treatment of BGE significantly reduced passive cutaneous anaphylaxis induced by antidinitrophenyl IgE. BGE also inhibited compound 48/80‐induced histamine production from rat peritoneal mast cells in vitro. Furthermore, BGE inhibited the induction of IL‐4 and TNF‐mRNA in human leukemia mast cells by phorbol myristate acetate (PMA) and A23187. All of these findings point to BGE having antiallergic properties, most likely through inhibiting histamine release and cytokine gene expression in mast cells (Chong‐Neto et al., 2022; Crespo et al., 2021; Jing et al., 2016; Pan et al., 2021; Papadopoulou et al., 2021).

### 5.3 Anticancer activity

Due to their lifestyle, cancer is the leading cause of death in developed and developing countries (Jemal et al., 2011). One of the century's major global problems is the formulation of functional foods to prevent chronic diseases, including cancer. It was found that eating a variety of foods, including buckwheat in diet, was reported to have a lower risk of lung cancer (Shen et al., 2008). Flavonoids and polysaccharides (Zhu, 2020); lectins (Bai et al., 2015); and phenylpropanoids (Li et al., 2014; Zheng et al., 2012) in buckwheat effect of apoptosis, differentiation, and cytotoxicity in cancer cell lines under in vitro conditions. The ability of flavonoids in buckwheat to bind with metal ions effectively inhibits the peroxidation of lipids. It prevents cell damage due to its antioxidant and free radical quenching activity, resulting in cancer prevention (Hou et al., 2017). Buckwheat contains protease inhibitors, which stop leukemia cells from multiplying (Wang et al., 2014). Isoquercetin prevents cell proliferation and induces apoptosis (Li et al., 2017). Buckwheat contains micronutrients such as zinc, selenium, and cadmium, which act as an immune booster and fight against cancer (Wang et al., 2015).

### 5.4 Hepatoprotective activity

Reactive oxygen and nitrogen species, as well as a variety of chemicals, damaged the liver in mice and rats. Lee et al. (2017) reported that buckwheat‐based flavonoids and their extract from the tartary cultivar protect the liver against carbon tetrachloride and ethanol‐induced damage. On a molecular level, the buckwheat flavonoids reduced serum aspartate transaminase activities. They increased superoxide dismutase enzyme activity, decreased liver dysfunction and hepatic inflammation, and improved the antioxidative and anti‐inflammatory functions for hepatoprotection (Lee et al., 2017). The hepatoprotective effects of buckwheat were related to polyphenols such as rutin (Ruan et al., 2020). Buckwheat‐based flavonoids have an inhibitory effect on the malondialdehyde end product of liver lipid peroxidation, maintaining the structural integrity of cells and preventing the leakage of soluble enzymes from the liver cells to protect the body against hepatotoxicity (Li et al., 2017).

### 5.5 Antidiabetic activity

Diabetes is a severe public health problem today, affecting over 300 million people (Zhang et al., 2011). Buckwheat helps avoid diabetes and its complications by reducing fasting blood sugar, increasing insulin levels, lowering glycosylated hemoglobin and glycosylated serum protein, and suppressing blood sugar levels (Lee et al., 2017). Buckwheat‐based products have significantly reduced blood sugar concentration and a lower risk of diabetes mellitus. Buckwheat should be cultivated more extensively as a grain crop due to its ability to effectively reduce the diabetes mellitus rate in people more prone to it. Both digested and undigested buckwheat flavonoids have improved glucose consumption and glycogen amount (Ruan et al., 2020). Rutin inhibits the glucosidases and amylase enzymes and thus reduces the glucose uptake in the small intestine by a decrease in carbohydrate digestion (Jadhav & Puchchakayala, 2012). Buckwheat starch has a greater sensitivity to digestive enzymes because of the structure and compactness of its granules (Zhu, 2016). Polyphenolic compounds, dietary fiber, and other nonstarch elements lead to the food matrix effects and lower the glycemic index of buckwheat‐based products (Singh et al., 2010). Fagopyritols are a special active ingredient of buckwheat‐soluble carbohydrates, used as a treatment for people with diabetes, noninsulin dependence, and polycystic ovarian syndrome.

### 5.6 Anti‐inflammatory and Antifatigue effects

Inflammation is a natural biological reaction to tissue damage, microbial pathogens, and chemical irritants (Pan et al., 2010), and their chronic stage is related to cancer development (Chen et al., 2018). An extract of buckwheat can lower inflammatory mediators, including interleukin‐6, monocyte chemoattractant protein‐1, tumor necrosis factor, and inducible nitric oxide synthase, as well as nitric oxide resulting in its inhibiting inflammation reaction (Li et al., 2017; Nam et al., 2017; Zhang et al., 2018). Different phenolic acids, such as feluric and p‐coumaric, in buckwheat decreased lipopolysaccharide‐induced inflammation activity (Hole et al., 2009). Fatigue is a term that encompasses a wide range of medical disorders related to pathology, general health, and physical activity. Exercising for a long time at a high intensity induces fatigue. Buckwheat protein significantly increased climbing, swimming time, and liver glycogen level, effectively reducing the blood lactate and serum urea contents (Jin & Wei, 2011).

### 5.7 Antihyperlipidemia activity

A diet with high cholesterol level triggers oxidative stress and raises the level in the blood leading to low‐density lipoproteins upregulation and the development of chronic diseases like atherosclerosis (Azorín‐Ortuño et al., 2012). Buckwheat provides a protective role against cardiovascular diseases, which could be related to its ability to modulate cholesterol levels, as Zhu (2016) reported in vitro and in vivo conditions. Buckwheat contains fantastic acids and flavonoids, which improve high‐density lipid levels in humans. Polyphenols and proteins in buckwheat inhibit blood vessel hardening and improve their integrity. The anthocyanin pigments in buckwheat act as antioxidants, helping prevent lipid‐based accumulation and end products in blood vessels (Zhang et al., 2017). Bran extract from buckwheat can also lower the lipid in the blood and liver, boosts antioxidants, and prevents peroxides in the blood (Ruan et al., 2020).

### 5.8 Antihypertensive activity

Hypertension affects approximately 1 billion people in the global population and is expected to be 1.56 billion by 2025 (Devos & Menard, 2019). The biological system of renin–angiotensin maintains blood pressure. The system involves the conversion of angiotensinogen into angiotensin I and then converted into angiotensin II by angiotensin I‐converting enzyme (ACE), resulting in hypertension (Jao et al., 2012). The presence of ACE inhibitors blocks the conversion reaction of ACE I to ACE II and hence results in lowering the pressure in blood vessels. The hypotensive effect of buckwheat was also supported both in in vitro and in vivo conditions as ACE inhibition (Jin et al., 2022). The buckwheat‐based flavonoids, particularly rutin, prevent blood vessel hardening, boost microcirculation, detoxify the blood, improve blood circulation, remove toxins, and lower blood and urine sugar levels (Hou et al., 2017). By assisting in regulating vasoconstriction and diastole, buckwheat extract helps lower blood pressure with quercetin as the key active component in minimizing oxidative stress of blood vessels and restoring vasodilation in clinical trials (Gimeńez‐Bastida et al., 2015).

### 5.9 Antineurodegenerative effect

Accumulating excessive protein (amyloid‐b protein) aggregation contributes to oxidative stress in the central nervous system, leading to neurodegenerative disorders (Citron, 2004). The most common neurodegenerative disorders are Parkinson's disease and Alzheimer's disease, all of which are triggered by genetic and environmental factors (Agnihotri & Aruoma, 2020). In vitro studies have shown that extracts from buckwheat seeds and plants have neuroprotective properties and antioxidant activities of acetylcholinesterase, butyrylcholinesterase, and tyrosine kinase (Gulpinar et al., 2012). Recent research suggests that neuroprotective effects in the buckwheat parts and their extracts were attributed to rutin (Choi et al., 2015).

### 5.10 Antigenotoxicity

Genotoxicity is a term used to describe a negative impact on the integrity of a cell's genetic material (DNA and RNA). Buckwheat extracts showed good protection against DNA damage induced by hydroxyl radicals under in vitro chemical assays. The use of buckwheat extract to repair the DNA damage was found to repair by over 50% owing to higher levels of phytochemicals and their ability to scavenge hydroxyl radicals and chelate iron (Cao et al., 2008). The DNA‐protective properties of buckwheat using a human hepatoma cell line reported that inhibition of DNA damage in the body was related to antioxidants from buckwheat extract containing rutin and quercetin (Vogrincic et al., 2013). The presence of rutin and quercetin components in buckwheat extracts works synergistically to protect against DNA damage (Wang & Zhu, 2015).

## 6 DEVELOPMENT OF GLUTEN‐FREE PRODUCTS FROM BUCKWHEAT

Gluten intolerance is an autoimmune disease caused by the consumption of cereal‐based gluten proteins that leads to mucosa damage in the small intestines through interaction among celiac patients, gluten diet, and response from the immunological system (Mir et al., 2018). Celiac disease involves loss of intestinal villi, incomplete digestion, and absorption of nutrients, affecting the overall function of the human body (Kreutz et al., 2020). The action of celiac responses involves gluten peptides produced by incomplete digestion, the deamidation process of glutamine residue by transglutaminase tissue in lamina propria of the small intestine, binding with antigen‐presenting cells and activated CD4+ T cells, and develops immune responses related to celiac diseases (Lindfors et al., 2019). According to the global gluten‐free products market (2021), the gluten‐free product market in 2019 was raised to USD 22 billion and is expected to reach USD 36 billion by 2026. The global gluten‐free products market is expected to grow at a compound annual growth rate (CAGR) of 8.5% from 2020 to 2027. The incidence of celiac disease has increased an average of 7.5% per year over the past several decades, as per the meta‐analysis by King et al. (2020), with their incidence rate highest in females and children. The pooled global prevalence of the celiac disease has been reported at 1.4% based on serologic tests, with prevalence values at 0.4% in South America, 0.5% in Africa and North America, 0.6% in Asia, and 0.8% in Europe and Oceania (Singh et al., 2020). The buckwheat‐based gluten‐free products are expected to grow at a CAGR of 4.2% and reach a market value of around US$ 837.3 M up to 2027. Celiac patients with a gluten content of 410–50 mg in the diet are very effective for developing immune responses causing damage to the gastrointestinal tract (Herman, 2012). The medication for celiac patients is shifting the diet pattern toward gluten‐free diets. The buckwheat‐enriched products are a recommended diet for people with celiac disease, with an excellent nutritional profile and good nutraceutical potential. The biologically active compounds in buckwheat gluten‐free products and their chemical structure are shown in Table 3. The increased interest in the health‐promoting properties of buckwheat focused consumers on being included in their diet as gluten‐free food. The consumer has used different approaches to increase the bioavailability of buckwheat‐based bioactive components. The consumer practices, including enrichment of cereal and legumes with buckwheat flour and pretreatment of buckwheat flour through enzymatic, germination, roasting, and fermentation, used for buckwheat‐based product formulation attract consumers with highly bioaccessible bioactive components. The bioactive components such as peptides, polyphenols, flavonoids, and antioxidants were significantly influenced by enzymatic, germination, roasting, and fermentation practices (Diowksz & Sadowska, 2021). The enrichment of buckwheat flour with added cereal, pulses, fruit, and vegetables has increased the bioactive components and their stability in buckwheat‐based products (Azad et al., 2021).

**TABLE 3:** Buckwheat‐based gluten‐free products and their biologically active compounds and chemical structure

Biologically active compounds	Chemical structure	Buckwheat gluten‐free products	References
Rutin	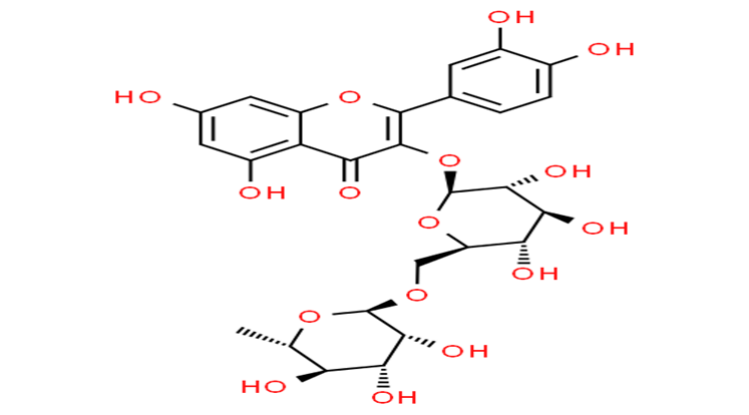	Buckwheat sprouts Fermented buckwheat sprouts Buckwheat bread Buckwheat noodles Buckwheat tea	Glavač et al. (2017), Suzuki et al., 2015), Szawara‐Nowak et al. (2014)
d‐fagomine	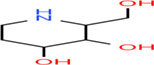	Buckwheat beer Buckwheat cookies Buckwheat dry noodles Buckwheat bread	Amezqueta et al. (2012)
Cycloartenol	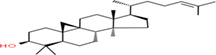	Broken roasted Buckwheat groats, Roasted buckwheat hulls, Buckwheat bran Whole roasted buckwheat groats	Dziadek et al. (2016)
Vanillic acid	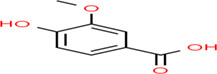	Buckwheat bread Buckwheat tea Buckwheat honey	Dziedzic et al. (2018), Szawara‐Nowak et al. (2014), Deng et al. (2018)
p‐Hydroxy‐benzaldehyde	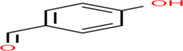		
p‐Coumaric acid	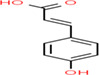		
Ferulic acid	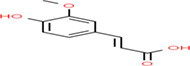		
Swertiamacroside	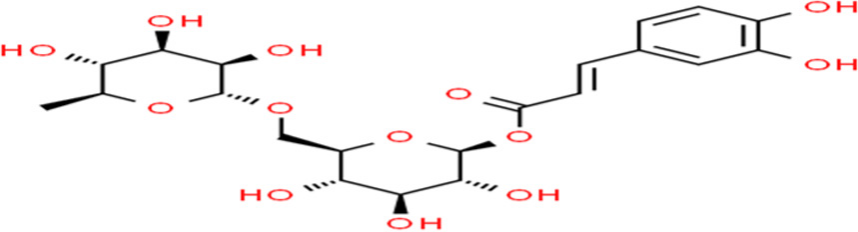		
Vitexin	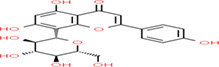		
Hyperin	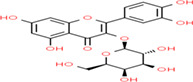		
Epicatechin	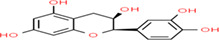		
Quercetin	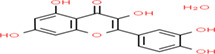		
Fagopyrin	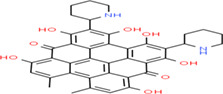	Buckwheat bread Buckwheat tea	Qin, Li, et al. (2013), Qin, Wu, et al. (2013), Glavač et al. (2017)
Fagopyritol	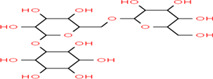	Buckwheat‐based biscuits Buckwheat groats Buckwheat sprouts	Zieliński et al. (2019), Izydorczyk et al. (2014)

### 6.1 Challenges and achievements in the technology of gluten‐free products

Buckwheat's utilization in developing gluten‐free products enhances its valorization in the gluten‐free market. It mitigates various diet‐related diseases and alternates gluten‐free diets to feed 1.4% of the world population (Bastida et al., 2015). The promising health ingredients and absence of gluten proteins in buckwheat focused on its use in food processing for various global gluten free buckwheat products (Małgorzata et al., 2018). There is an increased demand for gluten‐free products since the incidence of celiac diseases or other gluten intolerance or allergies. People with celiac disease have to restrict their diet of gluten proteins and shift their diet to gluten‐free products (Rosida et al., 2022). To reduce the prevalence of celiac disease and ensure customer‐acceptable quality, cutting out gluten from the diet presents technological obstacles. Gluten is the abundant structural protein complex found in wheat and exhibits the technofunctional properties of wheat‐based products (Allai et al., 2022; Sapone et al., 2012). The elimination of gluten in food products results in defects that appear in the form of quality attributes, nutritional characteristics, and consumer acceptance. Hence, the development of gluten‐free products for celiac patients is unrealistic to mimic the overall qualities of gluten products. Different approaches and technologies were adopted to overcome gluten‐free products' defects and make them acceptable to celiac patients. Incorporating nutritional ingredients, hydrocolloids, and enzymes to modify and mitigate the defects and problems of gluten‐free products (Alvarez‐Jubete et al., 2009; Hamada et al., 2013; Ronda et al., 2015). Other than altering formulations, technologies like high pressure, extrusion, and sourdough fermentation acting directly on the product's base material also bring a promising result, mimicking the gluten product qualities.

### 6.2 Use of buckwheat in the technology of gluten‐free products

The buckwheat was used to prepare different gluten‐free products such as bakery products (bread, biscuits, and cookies), noodle making, tea preparation, and extruded products with good organoleptic quality and consumer acceptability.

#### 6.2.1 Gluten‐free bakery products

In the bakery industry, there is a great scope to create innovative and health‐promising products by utilizing bioactive‐rich ingredients to produce functional bakery products. The buckwheat flour with bioactive‐rich components positively impacted human health (Zielinska et al., 2010). Subsequently, the exploration of bakery products (bread and biscuits) from different functional ingredients, modification, advancement in final product functionality, and composite flour‐based bakery products (bread, biscuits, and snacks) increases the consumer demand for these products. Buckwheat‐based ingredients in enriching bakery products are a new gluten‐free food with nutraceutical attention. The incorporation of buckwheat flour ingredient in bread development enhances the nutritional quality of bread with an increase in nutrients such as protein and mineral content (Wronkowska et al., 2013). The addition of buckwheat flour in product development leads to a technological problem in bread development due to its viscoelastic properties, lower baking quality, and acceptability by consumers (Hager et al., 2012; Saturni et al., 2010). The characteristics of gluten‐free bread, such as loaf volume and crumb texture, improved significantly with the incorporation of buckwheat as compared to control gluten (Alvarez‐Jubetea et al., 2010). Besides nutritional quality, the incorporation of buckwheat in bread enhances the antioxidant activity, low glycemic index, and functional properties of bread (Wolter et al., 2013; Wronkowska, Zielinska, et al., 2010; Wronkowska, Soral‐Śmietana, et al., 2010). The sensory and quality of buckwheat‐based bread were improved by incorporating other nonbuckwheat‐based ingredients such as starch, corn flour, and rice (Torbica et al., 2010; Wronkowska et al., 2013). The development of bread from buckwheat needs more effort in terms of technological and formulation aspects to enhance the overall quality and alternate targets for celiac diseases with high consumer acceptability. The buckwheat flour incorporation for the development of gluten‐free products enhances their nutritional and technofunctional properties. The addition of buckwheat flour in bread development improved the bread quality, protein, dietary fiber, mineral content, and textural properties (Krupa‐Kozak et al., 2011). The utilization of buckwheat flour in bread preparation enhances the resistant fiber, proteins, shelf life of bread, and good consumer acceptability due to the umami taste and characteristic aroma of buckwheat flour (Suzuki et al., 2020). The increase in buckwheat flour the bread development increased their rutin and antioxidant activity (Vogrincic et al., 2013). The gluten‐free‐based buckwheat bread reported a high level of protein, phenolic content, and antioxidant activity compared to amaranth and quinoa flour‐enriched gluten‐free bread (Chlopicka et al., 2012). The buckwheat, along with other pseudocereals, is considered an alternative to gluten protein‐based bread, with an increased nutritional profile and phenolic content (Schoenlechner et al., 2010). Wronkowska, Soral‐Śmietana et al. (2010) and Wronkowska, Zielinska et al. (2010) formulated a buckwheat‐based gluten‐free bread with corn starch as the base material. They showed increased activity of antioxidants, minerals, proteins, and vitamins compared to control bread without buckwheat flour. Furthermore, bread containing whole‐grain buckwheat flour expressed higher antioxidant and phenolic compounds (Sakac et al., 2011). However, the addition of buckwheat in bread development, besides the increase in nutritional content and functionality, is restricted due to their low baking quality and consumer acceptability (Saturni et al., 2010).

Cookies and biscuits from buckwheat are bakery products with health‐promising functional ingredients for gluten‐intolerant people (Sedej et al., 2011). Cookies made from buckwheat proved to have excellent product quality and acceptability by consumers by up to 20% (Torbica et al., 2010). Gluten‐free cookies from buckwheat with added chickpea flour enhance nutritional value and organoleptic properties compared to control wheat‐based cookies (Yamsaengsung et al., 2012). The area of gluten‐free cookies needs much more studies for the optimization process to enhance the quality and sensory properties for targeting celiac patients as a diet to control the impact of celiac diseases. The buckwheat‐enriched snacks, not less than 30 percent buckwheat flour with corn flour, showed good acceptability as an attractive appetizer with high nutritional properties (Wojtowicz et al., 2013). The biscuits prepared by incorporating buckwheat changed the physicochemical and organoleptic properties and increased spread, hardness, and fracturability (Filipcev et al., 2011). The incorporation of buckwheat from 20% to 50% enhances the sensory attributes, biofunctional properties, protein, fiber, micronutrients, polyphenolic content, and antioxidant activity (Baljeet et al., 2010; Filipcev et al., 2011). The cookies from buckwheat are also gluten‐free products with broad consumer acceptability and nutraceutical properties. The cookies made from buckwheat flour were more protein and fiber rich than those from wheat flour (Sedej et al., 2011). The protein content of the cookies formulated from buckwheat flour was in the range 4.34%–5.45%, fat (18.81%–20.04%) and the fiber content 0.39%–0.68%, with better cookie qualities (Altındag et al., 2015).

#### 6.2.2 Gluten‐free noodles and pasta

The noodles are convenient, easy to prepare, acceptable by consumers, and nutritionally rich products (Sofi et al., 2019). These noodles and pasta properties would be acceptable for celiac patients to develop gluten‐free products. The buckwheat noodles, commonly called soba noodles, are prepared by substituting with cereal flour or with buckwheat flour only (Hatcher et al., 2011). Buckwheat noodles were prepared with other ingredients such as green tea powder, mushroom, or seaweed to enhance sensory and texture quality (Yoon et al., 2007). The textural properties of noodles are an important quality parameter for judging the sensory score of the noodle and are dependent upon the amount of starch, protein, and fiber content present in noodles (Hatcher et al., 2011). The demand for noodles has increased from buckwheat due to its nutraceutical potential, but consumers' sensory quality is only partially acceptable due to a lack of a viscoelastic network (Han et al., 2012). Recent studies on noodles prepared from buckwheat were done to improve texture, sensory, and noodle quality to make acceptable noodles for gluten‐intolerant people (Bouasla & Wójtowicz, 2019).

Buckwheat‐based noodles are known as soba‐type noodles, with 35% buckwheat flour having good texture and cooking qualities (Hatcher et al., 2011). Buckwheat‐based noodles also contain 60%–100% of buckwheat flour for preparing soba noodles with good consumer acceptability (Sun et al., 2019). Buckwheat noodles have been added with functional ingredients such as green tea, shiitake mushroom, or seaweed powder to enhance their nutraceutical potential (Yoon et al., 2007). The increase in demand for buckwheat‐based noodles is an alternative product for gluten‐intolerant people with a good source of nutrients. However, the nongluten protein in noodle dough does not produce a cohesive structure that affects the textural qualities (Hatcher et al., 2011). The buckwheat flour incorporation in gluten‐free noodle development increased the mineral and nutritional composition. However, buckwheat addition to noodles reduced the cooking quality and color parameters. The noodles produced from the fermented buckwheat increase the amount of amino acids and minerals, whereas reduce the allergenic proteins and phytic acid (Bilgicli, 2009).

#### 6.2.3 Other gluten‐free products

The buckwheat, rich in bioactive compounds, can also be processed into tea, beer, and extruded products to enhance the valorization of buckwheat beyond bakery products. The process of tea formation from buckwheat involves many steps to minimize the bioactive compound degradations. The process involves soaking, steaming, and drying of buckwheat seeds, and then dehulled seeds are roasted and powdered into tea development (Qin et al., 2010). The effects of this thermal processing on the nutrient composition and polyphenolic content depend upon the cultivars and processing time. The thermal stability of chemical constituents such as proteins in buckwheat tea is related to the proportion of lipid content in the buckwheat seed (Jin et al., 2022). The alternate method of retaining the nutrients, polyphenols, and antioxidants is using microwave heating for buckwheat tea preparation (Zhang et al., 2012). With a nutraceutical potential, Buckwheat tea is used in most Asian and European countries (Zielinska et al., 2013). The presence of rutin in buckwheat plant parts such as flowers and leaves was used for the preparation of tea, and the processing of these buckwheat‐rich rutin plant parts into tea showed a slight change in rutin content during boiling (Xu et al., 2019). Moreover, buckwheat's byproduct, such as hulls rich in flavonoids, was used to prepare infusions or teas (Zielinska et al., 2013). Buckwheat has been used in the production of malt as a basis of a mash for the development of beer suitable for celiac sufferers or others sensitive to specific glycoproteins (Agu et al., 2012).

Buckwheat has been shown as an alternative source of gluten‐free beer due to its dense nutritional profile (Brasil et al., 2020). In recent years, the investigation into the use of buckwheat in the production of beer has increased to avoid gluten‐based grains for beer development and the presence of a high concentration of amylases in germinated buckwheat grains and other water‐soluble compounds (Agu et al., 2012). The fermented beer prepared from buckwheat malt showed an increase in free amino acids, nitrogen, and minerals, whereas a decrease in antinutritional factors (Nic Phiaris et al., 2010).

Extrusion technology in food processing is used to produce food products with broad diversification, product quality, and consumer acceptability. The extruded products include pasta, modified flours, textural vegetable protein, meat analogs, snacks, and starch‐based food (Leonard et al., 2020). The extruded products have good digestibility and consumer acceptability and are available in various shapes and sizes due to the extruder's high pressure, mixing, and shear operations (Alam et al., 2016). The end‐user consumers' preference for healthy foods focuses on the extrusion technology industry to shift functional extruded products with added fiber, resistant starch, antioxidants, and vitamins (Chillo et al., 2010; Leonard et al., 2020). The buckwheat pseudocereal with nutritive dense, rich ingredients focused the researchers on formulating extruded products from buckwheat and was reported as prebiotic and maintains gut microbiota and reduced cholesterol level (Petrova & Petrov, 2020). The extruded products from buckwheat maintain nutrients during extrusion processing with higher antioxidant activity than roasted buckwheat. The protein digestibility, dietary fiber, and polyphenols in extruded buckwheat products are retained in higher amount, suggesting the addition of buckwheat flour as a functional supplement for the production of functional extruded products (Klepacka & Najda, 2021). The pasta produced from extrusion technology is an easy‐to‐prepare and available food product in the market with high acceptability by consumers. Buckwheat flour used in pasta maintains good‐quality dough and texture without affecting the cooking qualities of gluten‐free pasta (Schoenlechner et al., 2010). Optimization for the development of gluten‐free pasta from buckwheat flour was used to produce better firmness, a good structural network, and improved cooking and sensory quality (Gao et al., 2018; Verardo et al., 2011).

## 7 CONCLUSION

Buckwheat is the main pseudocereal with an excellent nutritional profile, rich in phytochemicals, vitamins, and minerals. Buckwheat is a cheap source of protein, with protein content higher than cereals would be an approach to mitigating protein‐related malnutrition in developing countries. The buckwheat associated with bioactive components has health and nutraceutical significance. The bioactive components isolated from buckwheat can be used in the pharmaceutical industry to treat various health‐related diseases. Nowadays most attractive trend in the food industry is the formulation of functional food with health benefits. In recent years, buckwheat‐related food products with good sensory and technofunctional qualities attract the food market with health benefits and are suitable food for people with gluten intolerance. However, more research and development are needed to improve gluten‐free buckwheat products' organoleptic.

## CONFLICT OF INTEREST

The authors declare no conflict of interest

## ETHICAL APPROVAL

The study involved no experimentation with human subjects.

## CONSENT TO PARTICIPATE

The authors declare their consent to participate in this article.

## CONSENT TO PUBLISH

The authors declare their consent to publish this article.

## Data Availability

Not applicable.
